# A community effort to protect genomic data sharing, collaboration and outsourcing

**DOI:** 10.1038/s41525-017-0036-1

**Published:** 2017-10-27

**Authors:** Shuang Wang, Xiaoqian Jiang, Haixu Tang, Xiaofeng Wang, Diyue Bu, Knox Carey, Stephanie OM Dyke, Dov Fox, Chao Jiang, Kristin Lauter, Bradley Malin, Heidi Sofia, Amalio Telenti, Lei Wang, Wenhao Wang, Lucila Ohno-Machado

**Affiliations:** 1 UCSD Health Department of Biomedical Informatics, University of California San Diego, La Jolla, CA 92093 USA; 2 Computer Science and Informatics, Indiana University, Bloomington, IN 47408 USA; 3 GeneCloud, Intertrust, CA, Sunnyvale, CA 94085 USA; 4 Centre of Genomics and Policy, Department of Human Genetics, McGill University, Montreal, QC H3A 0G4 Canada; 5 School of Law, University of San Diego, San Diego, CA 92110 USA; 6 Cryptography Group, Microsoft Research, San Diego, CA 92122 USA; 7 Department of Biomedical Informatics, School of Medicine, Vanderbilt University, Nashville, TN 37203 USA; 8 National Human Genome Research Institute, Rockville, MD 20894 USA; 9 The J. Craig Venter Institute, La Jolla, CA 92093 USA

## Abstract

The human genome can reveal sensitive information and is potentially re-identifiable, which raises privacy and security concerns about sharing such data on wide scales. In 2016, we organized the third Critical Assessment of Data Privacy and Protection competition as a community effort to bring together biomedical informaticists, computer privacy and security researchers, and scholars in ethical, legal, and social implications (ELSI) to assess the latest advances on privacy-preserving techniques for protecting human genomic data. Teams were asked to develop novel protection methods for emerging genome privacy challenges in three scenarios: Track (1) data sharing through the Beacon service of the Global Alliance for Genomics and Health. Track (2) collaborative discovery of similar genomes between two institutions; and Track (3) data outsourcing to public cloud services. The latter two tracks represent continuing themes from our 2015 competition, while the former was new and a response to a recently established vulnerability. The winning strategy for Track 1 mitigated the privacy risk by hiding approximately 11% of the variation in the database while permitting around 160,000 queries, a significant improvement over the baseline. The winning strategies in Tracks 2 and 3 showed significant progress over the previous competition by achieving multiple orders of magnitude performance improvement in terms of computational runtime and memory requirements. The outcomes suggest that applying highly optimized privacy-preserving and secure computation techniques to safeguard genomic data sharing and analysis is useful. However, the results also indicate that further efforts are needed to refine these techniques into practical solutions.

## Introduction

Rapid advances in sequencing technologies have enabled the meaningful use of human genomic data in a wide range of healthcare and biomedical applications.^1^ All of Us program, formerly known as Precision Medicine Initiative will generate genomic data, in combination with electronic health records and participant-reported data, from approximately one million US residents with diverse backgrounds.^2^ The availability of such data creates many exciting opportunities to accelerate scientific discovery, engineer better and targeted therapies for patients, and, ultimately, improve health. Given the large amount of genomic data, efficient sharing, proper storage and rapid processing are critical to reach such goals. However, various challenges have emerged in managing, sharing and processing large-scale human genomic data, as they may require extensive computing resources and cross-institutional collaborations that may raise privacy concerns.

Several studies have demonstrated the vulnerability of human genomic data if they are insufficiently protected: re-identifying patients from an ‘anonymous’ database,^3–6^ reconstructing allele frequencies for individuals,^7^ predicting predisposition to diseases,^3,7,8^ and even building a 3D face from human genomic data.^9^ As genomic information is shared among blood relatives, the improper disclosure of individual genomic data may affect family members’ privacy.^10,11^ Privacy concerns are further heightened when considering the irrevocable character of human genomic data once they are disseminated. As methods progress,^8,12^ new privacy threats are likely to emerge. For example, a new privacy risk from genomic data sharing (GDS) Beacons project^13^ was recently reported by Shringarpure et al.^8^ Beacons are web-based services that answer queries about allele presence, such as whether a specific nucleotide (e.g., T) exists in a data set for a specific genomic position (e.g., on chromosome 2 in position 12,345). Shringarpure et al. demonstrated that an individual can be re-identified by repeatedly querying the genome data sets via an open-access Beacon for alleles associated with an individual’s genome, with each query increasing the statistical confidence regarding the victim’s presence in the data set. Furthermore, genomic data from populations with rare diseases may have higher re-identification risk than those from populations with common diseases.^14^

In addition to existing technical strategies for protecting genome data privacy,^12,15–22^ several policies and regulations have been enacted. For example, the 2014 GDS Policy of the National Institutes of Health requires human genomic data to be de-identified^23^ before being shared. The GDS specifically indicates that de-identification should be accomplished by, at the very least, removing the 18 explicit and quasi-identifiers defined in the Safe Harbor method of the Privacy Rule for the Health Insurance Portability and Accountability Act of 1996 (HIPAA). However, as has been alluded to, various studies show that genomic data without explicit identifiers are still subjected to certain privacy risks.^5,7^ Therefore, it is necessary to better understand the limits of existing technical protections and continue to develop novel solutions to enhance privacy protection in human genomic data access, sharing, and analysis.^12,24^ To stimulate these efforts, we began organizing the annual Critical Assessment of Data Privacy and Protection (CADPP) competitions in 2014 to evaluate the state-of-the-art in human genome privacy protection and secure computation technologies.^19,20,25^ Here, we will review the first two CADPP competitions and then focus on the discussion of the current progress observed in the 3rd CADPP competition.

## Community efforts for protecting human genomic data privacy

Given the utility of human genome data and their sensitive nature, it is imperative to develop practical and rigorous privacy protection methods. Several recent surveys^12,24^ discussed relevant techniques. It remains unclear how well existing privacy protection techniques can be effectively applied to large-scale human genomic data. This happens because there is often a lack of direct comparison of different methods in real-world scenarios, which makes it difficult for researchers to understand their capabilities and limitations.

We organize competitions with open challenges to tackle emerging privacy issues that have direct impact on human genomic research. The organizing committee consults with human geneticists to carefully select tasks of broad interest. Members of the committee developed baseline algorithms to assess the feasibility of these tasks and clearly define the criteria for performance evaluation. In 2014, we organized the first CADPP competition^19^ to call for practical and privacy-preserving solutions based on the differential privacy^26^ framework for protecting the outcome of genomic data analysis. The best solutions showed encouraging results, with potential use in GWAS while providing provable privacy guarantees.^19,27,28^ The 2014 competition, however, did not address privacy and security issues of storage and computation, which are among the most critical when utilizing cloud computing services to conduct human genomic research. Thus, in 2015, we organized the second CADPP competition to solicit secure solutions on protecting genomic data analytics in the cloud.^20^ Despite the exciting progress demonstrated in that competition (e.g., certain secure solutions such as homomorphic encryption have been improved significantly), there remained many emerging problems (e.g., the emerging re-identification risk on the Beacon system^8^) that needed to be addressed, which motivated the third, and most recent instantiation, of the competition in 2016.

The third competition extended the scope to tackle three current genomic data privacy challenges in real world environments, Track 1 focused on hardening Beacons from detection of an individual’s presence in a data set. Track 2 focused on how to support privacy-preserving searches of patient genomic data across organizations. Track 3 focused on securing data resulting from genetic testing in a public cloud. We received a total of 17 solutions from 16 teams in 7 countries. A full list of the participating teams can be found on the 2016 CADPP competition website.^25^ A two-member team from Vanderbilt University, a six-member team from IBM, Cornell University and Bar-Ilan University, and a seven-member team from Microsoft Research won Tracks 1, 2, and 3, respectively. In addition, more than 50 teams from 13 countries attended the competition workshop.

We believe both competitions and traditional paper publishing can further the advancement of the science of genomic privacy. Here, we take a moment to review advantages that competitions enable in promoting genomic privacy research. First, there are often gaps among different research communities (e.g., security, genetics, and bioethics) that focus on the topic of genomic privacy. For example, papers from the cryptography community tend to focus on technical contributions (e.g., advanced protection models) that may be ill-posed for real-world applications or neglect ethical or regulatory concerns that can be complemented by researchers from other fields. Without designing specific tasks in competitions, different published papers may focus on different use cases with different protection schemes or from different perspectives. Through the competitions, we can create benchmarks of the state-of-the-art solutions for researchers, policy makers and funding agencies. Therefore, one can gain a better understanding of the capabilities of the current technology available for protecting large-scale genomic data. Additionally, tasks involved in competitions are often tailored toward real-world biomedical applications through coordination with researchers and practitioners from different fields, which we believe helps in the prioritization of genome privacy research. Specialized scientific news outlets such as Nature News^29^ and GenomeWeb^30–32^ reported on these events, showing an increasing interest on genomic privacy protection in the biomedical community at large.

In the rest of this article, we will focus on the discussion of results and key findings of the competition. Accepted papers that describe the details of the solutions provided by teams can be found in a special issue of BMC Genomic Medicine focused on the competition.^33^ Since only a subset of the teams submitted papers to the BMC special issue, we also provide a link on our competition website^25^ to recordings of their presentations for readers who may be interested in the technical details.

## Track 1: Practical protection of GDS through beacon services

The international Beacon project was designed as a public web service to enable institutions to share summary information about genomic data repositories. Specifically, Beacon allows for users to query for the the existence of any genomes given the query inputs as variant, position and chromosome. Currently, there are more than 200 programs involved that contribute to the Beacon Network. However, Shringarpure and Bustamante^8^ (SB) demonstrated that, under the right circumstances, a malicious user could identify the presence of an individual behind a beacon through repeated queries of the individual’s genomic variants.

Given the vulnerability of such beacons, we designed the first challenge to solicit approaches to mitigate a modified SB model. For this challenge, we constructed a Beacon database of 500 genomes from the 1000 Genomes project.^34^ In the modified SB model, the allele frequencies derived from the 1000 Genomes project were utilized instead of a presumed distribution of allele frequencies in the original SB model. The evaluation of Track 1 was based on both the detection power and the utility of the solutions. More specifically, with a detection power no greater than 0.6 (in terms of the likelihood ratio test), we evaluated how much utility (in terms of the maximum number of correct responses through a series of random queries) could be preserved by the various solutions.

**Fig. 1:**
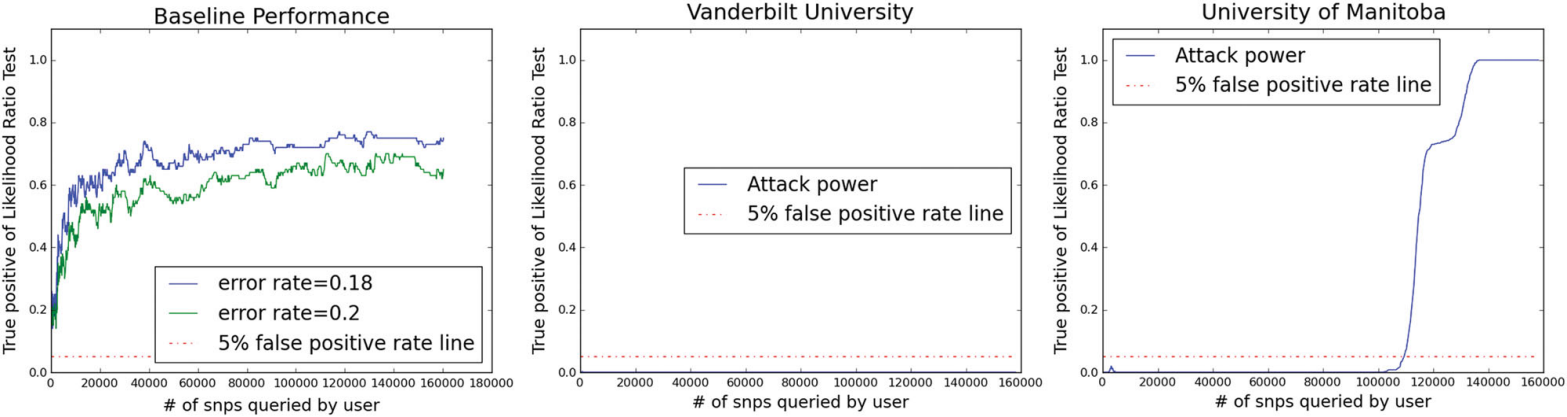
Performance of Track 1 in terms of detection power vs. the number of Beacon queries for the top two entries: Vanderbilt University (center) and University of Manitoba (right), as well as our baseline (left). The error rate is defined as the number of correct responses over the total number of queries issued by a malicious user

In our previous work,^35^ three different mitigation models were proposed: (S1) Beacon alteration strategy; (S2) Random flipping strategy; and (S3) query budget per individual strategy. However, we only include the results of the S2 models as our baseline performance for the 0.2 and 0.18 flip probabilities. We consider S2 as a more sophisticated version of S1 by flipping only a portion of the unique alleles. This results in a more fine-grained control between utility and privacy. As a consequence, we did not include S1 as the baseline during our evaluation. S3 was not chosen as a baseline in Track 2 because we assumed the beacon service does not keep track of the queries per individual. The performance of our baseline^35^ and performance of the top two teams, the first from Vanderbilt University^36^ and the second from the University of Manitoba,^37^ are depicted in the Fig. 1. The performance from both participating teams significantly outperformed our baseline. The winning solution from Vanderbilt was able to answer 160,000 queries without presenting the malicious user with any detection power. However, on the utility side, an error rate of 0.115 was observed over the 160,000 queries. The error rate is defined as (1 − # of correct response)/(# of queries).

## Track 2: Privacy preserving search of similar cancer patient across organizations

The motivation for Track 2 is to enable two institutions to jointly perform certain genomic analyses without directly sharing genomic data. The outcomes of this track demonstrated the feasibility of applying secure multiparty computation to this problem. This is important because patients with similar genomic variants might provide clues to the associated disease. This claim is justified by a recent *Science* paper^38^ that reported on applying secure multiparty computation to study common phenotypes of patients who share the same rare variants across two hospitals. In this track, we asked teams to develop SMC solutions for a scenario where privacy was required for coordination between two institutions. Specifically, one institution hosts a private database of patient genomes, while the other institution has a private genome from a single patient to compare against the database. The institutions aim to identify the top *k* most similar patients without leaking information other than the final results. Due to computational complexity concerns (e.g., execution time) based on our baseline implementation, the ZNF717 gene sequences with ~3470 bps encoding of a BRAB zinc-finger protein were used to query databases with 500 patients. The selection of such a data set ensures that most solutions could be evaluated within a few minutes. This was at the expense of an extensive evaluation involving longer genomic sequences and a larger number of records.

In Track 2, similarity was defined as the Levenshtein distance between two genomes. However, determining the exact distance is computationally expensive, so we allowed solutions to adopt any approximation methods to speedup the computation and preserve as much accuracy as possible. We assessed the solutions in terms of (1) accuracy (i.e., proportion of returned genomes that were truly in the top *k*) and (2) speed in computation and communication costs. We established a real-world environment with a private database and private query programs hosted at Indiana University (with a 4-core Intel(R) Xeon(R) CPU at 3.07 GHz and 4.03GB memory) and University of California at San Diego (with the secure configuration), respectively. We selected *k* equal to 1, 3, and 5 as benchmarks for the competition because to be in alignment with the typical risk assessment levels applied by privacy professionals.^39^ All results were averaged over 5 runs.

Table 1 summarizes the results of Track 2 from the participating teams. The solution from the IBM team 1 provided the best performance with a runtime under 12 s and an accuracy that implied the top k list was never off by more than one instance. During the workshop,^25^ the IBM team also demonstrated that their solutions were scalable to handle a larger database of 4000 patients. With respect to the privacy/security concern, each team had to provide a note that explained the underlying algorithms with at least a 80-bit security guarantee. The algorithms were peer-reviewed by security experts. In addition, the organizers have reviewed the submitted implementation. However, the potential risks due to implementation bugs were not considered during our evaluation. We rank solutions in the order of accuracy and speed with the constraint that the execution time should be no longer than 3600 s.

**Table 1:** Results for competition Track 2 (secure collaboration), where “Accuracy@*k*” is defined as the average of all correctly identified top *k* results over 5 runs using databases with 500 patients records

Team	Run time (s)			Accuracy@*k*		
	Top 1	Top 3	Top 5	Top 1	Top 3	Top 5
IBM Team 1	**11.37**	**11.41**	**11.62**	**1**	**3**	4
Indiana University at Bloomington^45^	209.03	273.14	337.79	**1**	**3**	4
University of Manitoba^46^	22.65	22.99	22.88	0	2	2
Cybernetica AS	80.97	67.47	64.64	**1**	1	1
University of Maryland	12.93	21	30.4	**1**	0.67	2.3
RWTH Aachen University	5700	>6300	>6300	**1**	**3**	**5**

The bold values indicate the best performance among teams

## Track 3: Testing for genetic diseases on encrypted genomes using public clouds

Cyber-infrastructure that has been developed for handling industry applications of big data (e.g., Open Science Grid, Amazon EC2, Microsoft Azure and Google Cloud) can be leveraged to manage, process and share large-scale genomic data in a sustainable manner. The NIH GDS policy^23^ states that genomic data downloaded from NIH databases can be processed in public clouds, but that the researchers and their institutions, as opposed to the cloud providers or NIH, are responsible for ensuring data security and privacy in such a cloud.^23^

The motivation for Track 3 was to develop novel solutions for securely outsourcing computation and storage of human genomic data to untrusted cloud environments. The outcomes of this track demonstrated that certain genomic analysis tasks can be efficiently evaluated over homomorphically encrypted data with task-specific optimization (e.g., data batching and hashing schemes). In Track 3, we allowed the participating teams to assume a semi-honest threat model, where the untrusted public cloud follows the protocol, but try to gain more information than allowed from the protocol. As an exemplary scenario, McLaren et. al.^40^ studied a real-world application for privacy-preserving genomic testing in the clinic, where 4149 variants from 230 HIV patients in Swiss HIV cohort study were homomorphically encrypted and outsourced to a storage and processing unit (i.e., an untrusted cloud). This study demonstrated the feasibility of searching on these encrypted data for ancestry inference and risk test computation.

The challenge in this track was to hide all data, query and access patterns from the cloud service provider about a genetic test. We specifically focused on the genetic testing case of Charcot-Marie-Tooth disease type 2I as it is associated with various single nucleotide variations according to the ClinVar database.^41^ We required participants to adopt homomorphic encryption to support long-term storage of the data and support a high level of security (at least 80 bits). The computation needed to be completed in one round of query and response and should retrieve less than 20 variants in each search. We instantiated the system to be a client-server model with an 10 Mbps network to resemble a typical cloud database, where the server has an Intel Xeon E3-1275v5 CPU at 3.6 GHz with 64GB memory. The performance of the proposed solutions were evaluated by computation time, storage space, and communication cost. Here, we consider the computation time as the primary metric in our evaluation. We prepared three different evaluation scenarios as follows: (1) one query with four variants against one VCF file with 10,000 records as a baseline performance for testing all solutions; (2) one query with four variants against one VCF file with 100,000 records to evaluate the scalability of the number of records for all solutions. (3) one query with one variant against 50 VCF files with 100,000 records to evaluate the scalability of both the numbers of patients and records for all solutions. However, due to page limits, we only report on the results of the second evaluation scenario. The detailed evaluation results for all scenarios can be found on our competition website.^25^ Table 2 summarizes the performance of Track 3 teams by querying four variants against one VCF file with 100,000 records on an average of 10 runs. The Microsoft team’s solution showed the best performance in terms of the fastest turnaround time for HME computation, results decryption and data transferring.

**Table 2:** A summary of the results for Track 3 (secure outsourcing)

Team	Data encryption time (s)	Encrypted data size (MB)	Secure computing time (s)	Result decryption time (s)	Total time (s) for computing, result decryption and transfer
Microsoft^42^	1.86	24.00	3.09	0.02	3.63
RWTH Aachen University^47^	34.90	255.00	15.28	0.68	16.32
EPFL^48^	137.60	147.00	6.79	9.28	19.26
Seoul National University^49^	51.02	10.00	21.10	0.005	25.11
IBM team 2	478.10	1660.00	959.10	200.70	1178.2
Waseda University	109.72	5447.82	8937.51	0.058	8938.81

## Discussion

In the competition, we engaged researchers from the human genomics and computer security communities to jointly study genomic privacy problems and provide novel solutions. We summarize the winning solutions as follows: (1) the winning team from Vanderbilt University proposed a strategic flipping method^36^ for Track 1. The key idea is to define the flipping strategy as an optimization problem that can maximize the utility (i.e., number of correct answers) and minimize the privacy risk (i.e., power of the attack). Furthermore, a greedy algorithm was adopted to search the flipping strategy space for a local optimum. (2) The IBM team provided a winning solution for Track 2 based on the idea of a reference-based partition strategy to approximate the Edit distance between two sequences. More specifically, the sequences from each institution were first aligned against a common public reference that was shared by the two institutions. Then, given a fixed block size of the reference genome, the aligned sequences were further partitioned. Finally, a secure aggregation over these block-wise Edit distances was applied to approximate the global Edit distance between the sequences. (3) The winning solution of Track 3 from the Microsoft team^42^ utilized a technique called permutation-based cuckoo hashing. This method improves the string-matching performance by shortening the strings that need to be homomorphically compared. This is accomplished by packing several queries together so that multiple queries can be evaluated under the same HME evaluation, and allowing batch-based SIMD (single instruction, multiple data) operations.

The latest competition demonstrated results with impressive performance, for example, supporting a secure Beacon service to answer 160,000 privacy-preserving queries with 88.5% accuracy, speeding up secure sequence similarity comparison over two distributed sequences (length > 1 million) to less than 15 s, and conducting homomorphic genetic testing on 100k records within 4 s. Many results were encouraging in that we observed advances on the order of several magnitudes in terms of computation overhead reduction in comparison to the previous year.

In particular, we note that the teams’ solutions were highly optimized with respect to the competition goals. Although many optimization techniques designed for the current competition tracks (e.g., data batching for SIMD computation in HME) can be extended to support other secure genomic data analysis applications, it remains infeasible to develop a universal secure framework that can support arbitrary analysis tasks. For example, data encrypted by a partial homomorphic encryption scheme can only support a certain number of accumulated homomorphic operations as a threshold, which limits their flexibility in reuse by other applications that may exceed the threshold without involving a re-encryption process.

For SMC, the competition track only considered a two-party scenario. Extending a solution to allow for more than two parties may result in significant computational and communicational overhead. As mentioned in the recently published Science paper by Jagadeesh et al.^38^, the scalability issues of secure two-party computation are considerable. We further identified limitations in the design of these competition tracks. For example, it is challenging to securely compute the exact edit (or Levenshtein) distance over long sequences without approximation. Advanced secure analysis tasks, like regression model learning, read mapping, and variant calling over encrypted data have yet to be considered in our competition. Given such limitations, we aim to develop a more extendable and flexible foundation for tackling the emerging privacy challenges in human genomic studies and close the technology gap in adopting these new technologies in practice.

We also engaged researchers from ethical and legal communities in the workshop. The competition produced positive results that show today’s current technology is capable of protecting the privacy rights of individuals when operating certain large-scale genomic data analysis services. As technology advances, researchers will be able to share genomic data on a large scale with very low risk of leaks of potentially identifying data or of breach of privacy regulations, such the HIPAA. Through this cooperation and participation in the activities of the Global Alliance for Genomics and Health (GA4GH), we aim to raise awareness of our technical solutions and promote their adoption through community standards such as the GA4GH Privacy and Security Policy and its Security Infrastructure Framework, which provides standards and implementation practices for protecting the privacy and security of shared genomic and clinical data.

Through competitions, privacy-preserving genomic data analysis models have demonstrated potential value with respect to the safeguarding of potentially sensitive information while supporting important studies. A recent *Science* paper^38^ by Jagadeesh et al. and a Genetics in Medicine paper^40^ by McLaren et. al. demonstrated the feasibility of using state-of-the-art models to derive genomic diagnosis without revealing patient genomes. Existing tools^38,40^ already make an impact on the genomic research community and our competition is calling for more efficient and scalable methods to address real world challenges. Over the last 3 years, we have witnessed significant progress and we, along with other groups around the world, including the Global Alliance for Health and Genomics, are working to get geneticists involved to improve such competitions. Specifically, the 2017 workshop is co-located with the American Society of Human Genetics annual meeting in Orlando to seek tighter collaborations between the two communities so we can engage geneticists and improve the competitions.

In the near future, we will focus on transitioning the outcomes from the competition into practice. For example, the solutions will have accessible interfaces (along with installation and user manuals) that allow integration into existing data-sharing portals (e.g., secure Beacon services, public cloud, etc.). We will also design more challenging tasks to tackle more practical problems in biomedical research, such as performing machine model learning over encrypted data, and adopting hardware based solutions^22,43,44^ to handle genomic data analysis at the whole genome scale.
